# The zebrafish subcortical social brain as a model for studying social behavior disorders

**DOI:** 10.1242/dmm.039446

**Published:** 2019-08-06

**Authors:** Yijie Geng, Randall T. Peterson

**Affiliations:** Department of Pharmacology and Toxicology, College of Pharmacy, University of Utah, 30 S. 2000 East, Salt Lake City, UT 84112, USA

**Keywords:** Autism, Phylogenetic conservation, Model organism, Social deficit, Neuropsychiatric disorders, Behavioral assay

## Abstract

Social behaviors are essential for the survival and reproduction of social species. Many, if not most, neuropsychiatric disorders in humans are either associated with underlying social deficits or are accompanied by social dysfunctions. Traditionally, rodent models have been used to model these behavioral impairments. However, rodent assays are often difficult to scale up and adapt to high-throughput formats, which severely limits their use for systems-level science. In recent years, an increasing number of studies have used zebrafish (*Danio rerio*) as a model system to study social behavior. These studies have demonstrated clear potential in overcoming some of the limitations of rodent models. In this Review, we explore the evolutionary conservation of a subcortical social brain between teleosts and mammals as the biological basis for using zebrafish to model human social behavior disorders, while summarizing relevant experimental tools and assays. We then discuss the recent advances gleaned from zebrafish social behavior assays, the applications of these assays to studying related disorders, and the opportunities and challenges that lie ahead.

## Abstract (precis)

**Summary:** The zebrafish has become an increasingly attractive model for studying social behavior disorders. This Review summarizes relevant methods, established social deficit models, emerging technologies and the anatomical basis of sociality.

## Introduction

Social behavior – defined as beneficial interaction between individuals in the same species – is essential for the survival and reproduction of social species, including humans and many other vertebrates. Social behavior involves specific behaviors such as conspecific preference, social communication, aggression and mating. Many, if not most, neuropsychiatric disorders are related to underlying social defects or are accompanied by social dysfunctions. These include autism, which is associated with deficits in processing social cues, and William's syndrome, which is characterized by an abnormally high enthusiasm for interacting with strangers. Other disorders that are not primarily social (e.g. schizophrenia and depression) may still interfere with normal social functioning. Therefore, developing and studying animal models with social deficits has far-reaching implications for many neuropsychiatric diseases, and studying these behavioral aspects requires developing specific behavioral assays.

Rodents are traditionally used to model disorders associated with social deficits. As highly social species, rodents possess many complex social behavior traits that mimic human behaviors. Additionally, researchers have established sophisticated protocols for studying these behaviors in rodents (Hanell and Marklund, 2014). These benefits make the rodents the current ‘go to’ models for studying disorders associated with social deficits. However, rodent models are not without drawbacks. They are expensive and labor intensive. They are predominantly nocturnal and highly sensitive to environmental disturbances such as light, sound, temperature changes and odors. Furthermore, they have not been very amenable to scalable or high-throughput assays. These drawbacks pose a limit to the broader application of these models in disease research.

In recent years, the zebrafish has rapidly become an attractive model for studying behavioral disorders. Adult colonies can be efficiently maintained at high density. Zebrafish give birth to large clutch sizes (>100 eggs per female for each round of breeding) and provide ample offspring for experimental manipulations. The embryos are small (∼0.7 mm in diameter) and develop *ex utero* with no special supplementations needed except for water during the first week of development, enabling easily scalable embryonic experimental perturbations. The transparent nature of zebrafish embryos during early development also facilitates imaging and analysis of developmental events. Recent advances in genome-editing technologies such as CRISPR can be applied to zebrafish embryos (Hwang et al., 2013). Unlike rodents, zebrafish are diurnal and can perform behavioral tasks under a normal light setting. Because they remain submerged in water during behavioral tests, zebrafish are not easily affected by minor environmental interferences such as weak sounds and smells.

**Fig. 1.**
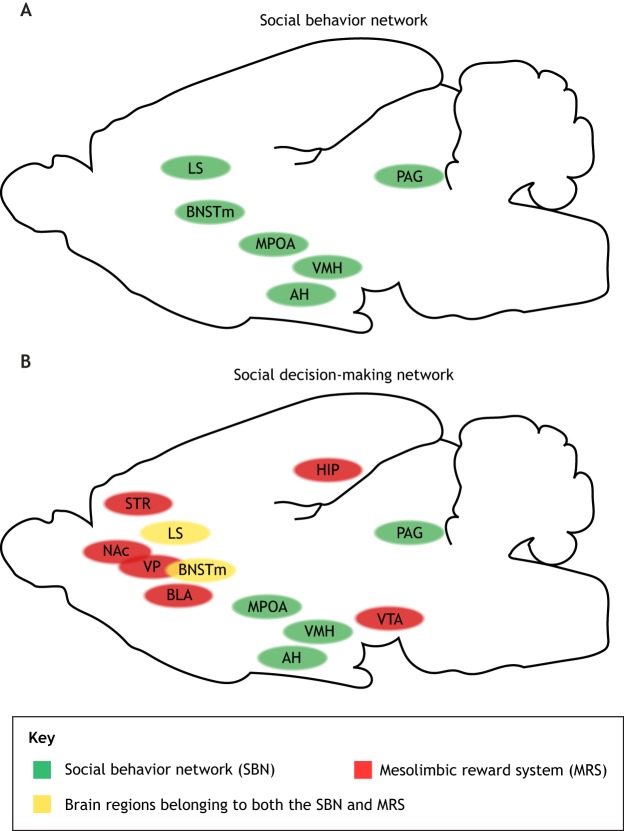
**Previous theoretical models of the social brain.** Brain structures that constitute previous models of social brain networks, illustrated in a mammalian brain from a lateral view. (A) The social behavior network (SBN) (Newman, 1999). (B) The social decision-making (SDM) network (O'Connell and Hofmann, 2011b), composed of the SBN and the mesolimbic reward system (MRS). AH, anterior hypothalamus; BLA, basolateral amygdala; BNSTm, medial bed nucleus of the stria terminalis; HIP, hippocampus; LS, lateral septum; MPOA, medial preoptic area; NAc, nucleus accumbens; PAG, periaqueductal gray; STR, striatum; VMH, ventromedial hypothalamus; VP, ventral pallidum; VTA, ventral tegmental area.

**Fig. 2.**
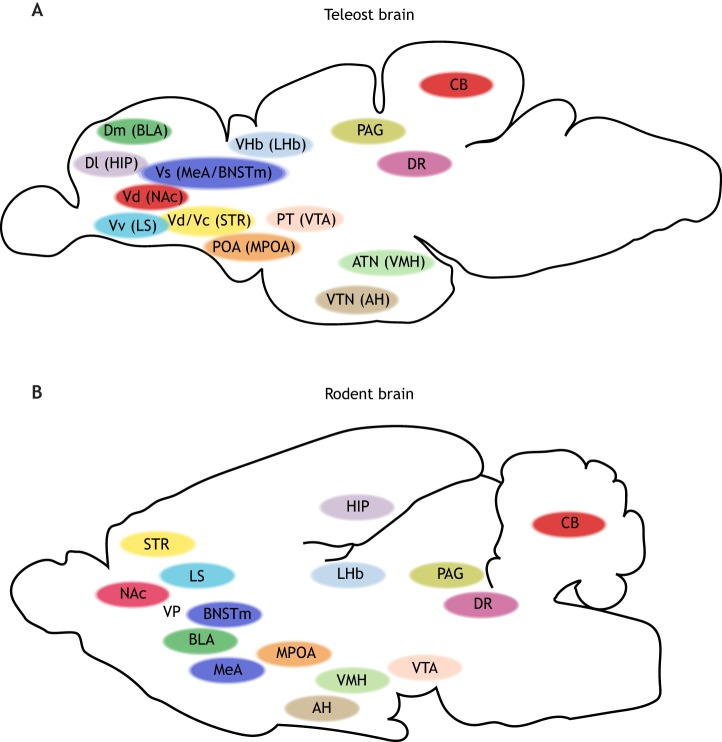
**The subcortical social brain (SSB) in zebrafish and mouse.** (A) The teleost SSB illustrated from a lateral view. (B) The mammalian (rodent) SSB illustrated from a lateral view. Areas with the same color mark regions that are homologous between teleosts and mammals. For regions with different nomenclatures between teleosts and mammals, the corresponding mammalian nomenclatures are appended after the teleost nomenclature in parentheses. AH, anterior hypothalamus; ATN, anterior tuberal nucleus; BLA, basolateral amygdala; BNSTm, medial bed nucleus of the stria terminalis; CB, cerebellum; Dl, lateral dorsal telencephalon; Dm, medial dorsal telencephalon; DR, dorsal raphe; HIP, hippocampus; LHb, lateral habenula; LS, lateral septum; MeA, medial amygdala; MPOA, medial preoptic area; NAc, nucleus accumbens; PAG, periaqueductal gray; POA, preoptic area; PT, posterior tuberculum; STR, striatum; Vc, central ventral telencephalon; Vd, dorsal ventral telencephalon; VHb, ventral habenula; VMH, ventromedial hypothalamus; VP, ventral pallidum; Vs, supracommissural nucleus of the ventral telencephalon; VTA, ventral tegmental area; VTN, ventral tuberal nucleus; Vv, ventral nucleus of the ventral telencephalon.

In this Review, we first discuss the neuroanatomical and neurophysiological evidence supporting the use of zebrafish to model human social behavior disorders (see Box 1; Figs 1, 2). We then describe the established experimental methods for studying social behavior deficits and examples of using these assays to model related human disorders. Finally, we explore relevant emerging technological advances and the opportunities and challenges that lie ahead in applying these technologies to social disorder modeling using zebrafish.

**Box 1. The ‘subcortical social brain’ and its evolutionary conservation between zebrafish and mammals**

Complex higher-order human social behaviors, such as face recognition, social cognition, perception of social signals, social judgement, social decision making and theory of mind, rely substantially on cortical input (Adolphs, 2003). The cerebral cortex is widely considered to be the major controller of these higher-order social behaviors (Adolphs, 2009; Blakemore, 2008, 2012; Frith, 2007). Human studies have identified specific cortical brain regions, such as the medial prefrontal cortex (mPFC) and the posterior superior temporal sulcus (pSTS), that contribute to these functions (Adolphs, 2009; Frith, 2007; Blakemore, 2008, 2012). This focus on cortical inputs sometimes overlooks the critical functions that subcortical brain regions play in regulating social behavior. In fact, a complex network of subcortical brain regions associated with social behavior exists and is highly conserved among all vertebrates (Newman, 1999; O'Connell and Hofmann, 2011b). Here, we conceptualize a ‘subcortical social brain’ (SSB) based on theoretical frameworks and recent experimental findings.

Originally suggested for mammals, Newman (1999) proposed a core ‘social behavior network’ (SBN) based on evidence from neuroendocrine and behavior studies. The SBN consists of several brain regions as ‘nodes’, including the medial amygdala, medial bed nucleus of stria terminalis, lateral septum, preoptic area, anterior hypothalamus, ventromedial hypothalamus and the midbrain periaqueductal gray/central gray (Fig. 1A). In this model, each node responds to a variety of social stimuli, and all nodes collaboratively respond with a distinct pattern to modulate different behavioral outputs.

O'Connell and Hofmann (2011b) pointed out the importance of the mesolimbic reward system (MRS), consisting of the ventral tegmental area, nucleus accumbens, basolateral amygdala, striatum, ventral pallidum, hippocampus and several regions overlapping with the SBN, in social behavior. They further argued that the SBN and MRS collectively constitute a larger social decision-making (SDM) network (O'Connell and Hofmann, 2012, 2011b) (Fig. 1B). Finally, they demonstrated that this network is largely conserved between zebrafish and mammals (O'Connell and Hofmann, 2011a, 2012). Functional analysis of immediate early genes after social interaction supports this hypothesized network (Teles et al., 2015).

Recent studies have linked additional subcortical brain regions to social behavior, and homologous structures for these socially relevant brain regions, such as the dorsal raphe (Dölen et al., 2013), lateral habenula (Golden et al., 2016) and cerebellum (Carta et al., 2019), are also present in zebrafish (Yokogawa et al., 2012; Amo et al., 2010; Heap et al., 2013). The anatomical and functional conservation of these SSB components are summarized in Table 1 and Fig. 2.

**Table 1.**
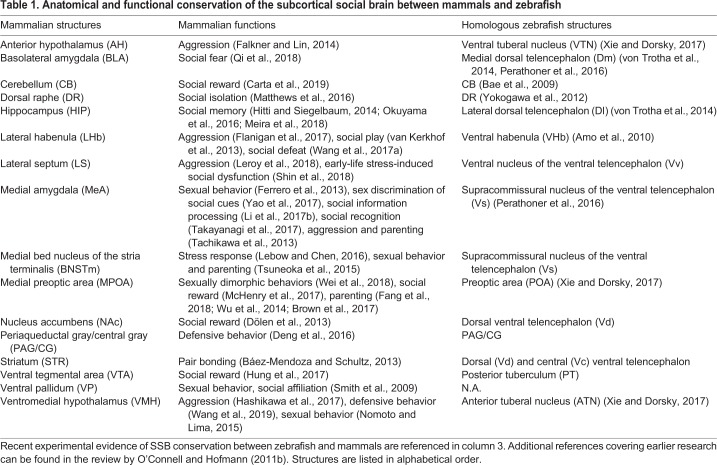
**Anatomical and functional conservation of the subcortical social brain between mammals and zebrafish**

Although many cortical regions in the mammalian brain are also relevant to social behavior by serving executive functions during social interactions and have been discussed elsewhere (Adolphs, 2009; Blakemore, 2008, 2012; Frith, 2007), we chose to focus this Review on the SSB for its relevance to zebrafish social behavior. We argue that its strong evolutionary conservation suggests a critical role in supporting the survival and reproduction of not only fish, but also other vertebrate species, including humans. Knowledge acquired from investigating the SSB may therefore provide valuable insights into human social behavior disorders (Lord et al., 2000).

## Zebrafish assays for studying social behavior and deficits

There are typically two approaches to modeling a behavioral disorder: by using an endophenotype (see Box 2 for a glossary of terms) assay or a behavioral assay. In this Review, we focus primarily on the behavioral approach.

**Box 2. Glossary**

**Alarm substance:** also known as Schreckstoff (startle/shock substance); a chemical alarm signal released by injured fish that induces fear in conspecifics. A method for extracting zebrafish alarm substance can be found in the report by Schirmer et al. (2013).

**Bayesian decision theory:** a statistical method that calculates the tradeoff between various decisions using Bayesian estimation.

**Chemogenetics:** the modification of biological macromolecules, such as proteins, to interact with previously unrecognized ligands. Engineered G protein-coupled receptors (GPCRs), such as designer receptor exclusively activated by designer drugs (DREADDs) (Armbruster et al., 2007), can be activated by an otherwise inert ligand to modulate the activity of genetically modified neurons.

**Delaunay triangulation:** for a given set of discrete points in a plane, a Delaunay triangulation generates a network of triangles between these points, which ensures that no point is inside the circumcircle of any triangle.

**Endophenotype:** an experimentally measurable trait that genetically segregates with an illness. Frequently used in psychiatric disease research to connect higher-order complex behavioral symptoms to genetics.

**Fetal alcohol spectrum disorders:** a group of conditions, including physical and behavioral problems, that can occur in a person prenatally exposed to alcohol.

**Fetal valproate syndrome:** a condition that can occur in a person by prenatal exposure to the anti-seizure medication valproic acid (sodium valproate).

**Immediate early gene:** a gene that is activated rapidly but often transiently at the transcription level in response to certain stimuli.

**Long short-term memory:** abbreviated as LSTM; a recurrent neural network architecture used in deep learning. It is particularly suitable for analyzing and making predictions for time-series data containing lags of unknown duration between important events.

**Morphant:** in zebrafish, gene expression levels can be knocked down at the early embryonic stage through embryonic injection of morpholino antisense oligonucleotides. A zebrafish treated with a morpholino to temporarily inhibit expression of a targeted gene is called a morphant.

***nacre*:** a zebrafish genetic mutation (Lister et al., 1999). Homozygous *nacre* mutants lack melanophores, a type of pigment cell, and are therefore partially transparent and visually different from wild-type zebrafish.

**Optogenetics:** an experimental method that uses light to control activation and/or inhibition of genetically modified neurons expressing light-responsive ion channels.

**Shannon entropy:** also known as ‘information entropy’; provides a measurement of the predictability of the value of a variable. In Eguiraun et al. (2018), Shannon entropy was used to measure the predictability of the trajectory of a shoal's centroid.

**Transfer entropy:** a measurement of the amount of directed transfer of information between two random processes. It provides a quantification of the cause-and-effect relationships between (possibly) coupled time series.

As larval zebrafish develop into adults, their behavior becomes increasingly complex. This is particularly pronounced for socially relevant behaviors. In this section, we briefly review several stereotypical social behaviors in zebrafish and discuss recent advances in developing assay platforms for studying these behaviors. We begin by describing assay setups for specific aspects of social behaviors using traditional and new experimental methods. We then discuss emerging technologies for social behavior analysis that will help improve the robustness, consistency and resolution of the current assay methods, including computer vision, machine learning, computational modeling, robotics, and virtual reality (VR) technologies.

### Social preference assay

Social preference behavior, or the innate tendency of an animal to observe, mimic and approach a conspecific, is well conserved among social vertebrate species. Often emerging early during ontogeny (Fantz, 1963), this simple and perhaps primitive form of social behavior forms a necessary foundation for the later, higher-order social functions such as shoaling, schooling and other complex social interactions. This behavior is routinely tested in rodents using a three-chamber social preference assay.

To study this behavior during development, it is desirable to design experimental systems that can pinpoint its earliest emergence. Hinz and de Polavieja (2017) discovered that zebrafish larvae start to show attraction toward a conspecific as early as 6-7 days post-fertilization (dpf). Although weak at this stage, the attraction quickly gets stronger each day during development. An important note for the experimental setup is that early larval zebrafish are also attracted to borders such as the wall of a Petri dish. Thus, to detect weak social attractions at the early developmental stage, a testing chamber with a deep center and gradually shallower edge may be used to counter this ‘border attraction’ by deterring the larvae from the border with shallow water.

**Fig. 3.**
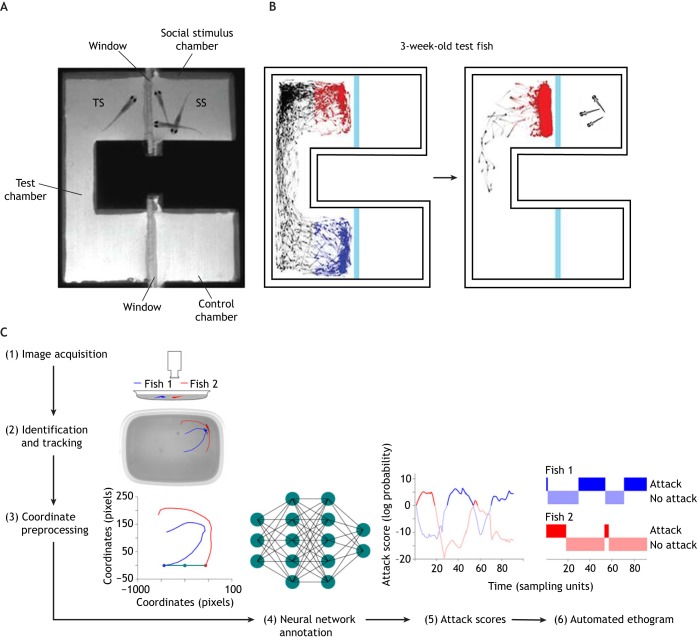
**Examples of zebrafish social behavior assays.** (A) The three-chamber social preference assay. Adapted from Dreosti et al. (2015), where it was published under a CC-BY licence. A test subject (TS) is placed inside a U-shaped chamber. Social stimulus (SS) fish are placed inside one of the chambers at the end of the U-shaped arena, separated from the test subject's chamber by a transparent window. The other end of the U-shaped arena is left empty as a control stimulus. (B) Three-week-old zebrafish develop a robust social preference. A test subject visits the two compartments randomly if both compartments are empty; if social stimulus fish are introduced into one compartment, the test subject is attracted to and interacts intensively with the social stimulus fish. Adapted from Dreosti et al. (2015), where it was published under a CC-BY licence. Red, movements in the social interaction zone; black, movements in the middle zone; blue, movements in the control zone. (C) Analysis of fighting behavior using machine learning. Adapted from Laan et al. (2018), where it was published under a CC-BY licence. Images are acquired (1) and two animals in the test arena are tracked individually (2). Fractions of the tracking coordinates are manually annotated for fighting behavior (3). This is then used to train a neural network (4), which automatically detects attacks by generating an attack score for each fish (5). An ethogram is generated based on the attack score (6).

Dreosti et al. adopted a design not unlike the three-chamber social preference assay for rodents and adult zebrafish (Dreosti et al., 2015): transparent windows divided a U-shaped test arena into three compartments, including a middle test compartment and two stimulus compartments (Fig. 3A). A test subject is placed inside the test compartment, and age-matched social stimulus fish are placed inside one of the two stimulus compartments, while the third compartment remains empty and thus stimulus free. The social preference of a test subject is quantified as the time it spends near the social stimulus fish. Two-week-old larvae exhibited a weak social preference, whereas, by 3 weeks, this preference behavior became highly robust (Fig. 3B). This system setup is simple to implement and does not require simultaneous tracking of more than one animal, but limits the test subject's input to visual cues, and prevents physical interactions between the fish.

Social preference behavior of adult fish is typically tested in larger three-compartmented tanks. Zebrafish of the same or different (Engeszer et al., 2004) strains, animated images of fish (Gerlai, 2017), 3D-printed fish models (Bartolini et al., 2016) or robotically controlled biomimetic zebrafish (Kopman et al., 2013; Ruberto et al., 2016, 2017) can be used as social stimuli. Researchers can place different stimuli in the two test compartments to assess preference. For example, wild-type (WT) fish typically prefer a WT conspecific over a *nacre* (Box 2) fish (Braida et al., 2012). Interestingly, this tendency is reversed by oxytocin, vasopressin, and amphetamine derivatives (Braida et al., 2012; Busnelli et al., 2016; Ponzoni et al., 2016). A WT test fish remembers a familiar fish from this assay for at least 24 h, and prefers to interact with an unfamiliar fish over a familiar one (Madeira and Oliveira, 2017). A two-compartment design has also been implemented for testing adult (Liu et al., 2018a) and juvenile (Patowary et al., 2019) fish, with social preference assessed by the proximity of a test subject to the social stimulus fish.

As will be demonstrated in examples in later sections, a three- or two-chamber social preference assay is frequently used to assess social preference of individuals subjected to different experimental treatments. A major benefit of this approach may be that test subjects are examined individually, as opposed to in groups as will be described in shoaling and schooling assays, such that the degree of social preference for each test subject can be easily quantified. A three-chamber assay setup also enables one to assess preference between two types of stimuli, such as two different fish strains. However, restrictions in sensory inputs and physical interactions limit the assay's ability to evaluate more complex modes of social behaviors.

### Shoaling

Groups of zebrafish naturally form compact aggregations, a behavior called shoaling, which emerges as early as 15 dpf (Hinz and de Polavieja, 2017). Benefits of shoaling may include better detection of and defense against predators, enhanced foraging and increased mating choices (Krause and Ruxton, 2002). While most studies choose the number of fish to be tested in a shoaling assay arbitrarily, a number that balances between minimizing animal usage and reducing variability may be estimated using a method based on Shannon entropy (Box 2) (Eguiraun et al., 2018).

Traditionally, shoaling is examined by two-dimensional (2D) video recording and analysis. A number of freely available computer vision programs and commercial software have been developed for detecting this behavior in grouped zebrafish (reviewed in Franco-Restrepo et al., 2019). Methods for quantifying shoaling behavior in a 2D recording typically don't require the fish to be individually distinguishable. Measurements of inter-individual distance and nearest-neighbor distance are commonly used to quantify the tightness of a shoal (Miller and Gerlai, 2007). Alternatively, analyzing a shoal by Delaunay triangulation (Box 2) can provide a unique measurement for the relative positions of each fish in a shoal configuration, as well as its overall tightness (Xiao et al., 2015). The trajectory of a shoal can be obtained by tracking its centroid (Eguiraun et al., 2018).

Because fish behave in a three-dimensional (3D) space, 3D recording and analysis systems are being developed using mirrors (Maaswinkel et al., 2013b; Audira et al., 2018b), multiple cameras (Macrì et al., 2017; Qian and Chen, 2017; Bishop et al., 2016; Al-Jubouri et al., 2017; Wang et al., 2017b; Butail and Paley, 2012) or a single camera with depth-sensing capability (Kuroda, 2018) to assess shoaling more accurately. The complex 3D trajectories of fish have been modeled using a long short-term memory (Box 2) network (Wang et al., 2017b).

WT zebrafish form tight shoals. As will be discussed in later sections, experimental perturbations can lead to changes in shoal cohesion. A reduction in shoal cohesion is often interpreted as decreased social interactions among members of the group. However, simple measurements of aggregation cannot fully reveal the complex, interactive and inter-dependent forces between individuals (Katz et al., 2011) or the collective dynamics of a group (Rosenthal et al., 2015).

### Schooling

In addition to shoaling, a group of zebrafish can ‘school’. While shoals are simple aggregations of individual fish, schools are shoals that exhibit polarized formations and synchronized motions. Density and group size affect shoal cohesion, but not polarization (Shelton et al., 2015). Acute treatment with alcohol strongly affects shoal polarization but only modestly inhibits cohesion, whereas nicotine significantly reduces cohesion but modestly affects polarization (Miller et al., 2013). These differences indicate that schooling and shoaling are two differentially regulated behaviors and that assessing both behavioral endpoints together may more effectively characterize the effects of experimental treatments. Tang et al. (2018) developed an unsupervised machine learning approach to examine schooling of adult zebrafish. Using this assay, the authors classified group behavior into distinct stereotypical states of polarization, and found that genetic mutations (see later sections for details) can alter the proportion of time spent or the tendency to transition between these states. While this approach provides an innovative way to quantitatively evaluate the propensities of a group to adopt stereotypical states of schooling, it is limited to detecting static patterns of group formation as a whole and cannot reveal dynamic interactions among group members.

### Aggression

Adult male zebrafish fight to establish dominance and hierarchy, and to compete for important resources such as food and mates (Huntingford and Turner, 1987). A simple way to assay aggressive behavior is by introducing a target for the test subject to attack. A mirror is often used to allow the test subject to attack its own reflection (Zabegalov et al., 2019). Alternatively, a dummy fish or a video recording of another fish can trigger aggression (Way et al., 2015). The number of times a test subject exhibits aggressive behavior, such as biting and charging, is counted to quantify its level of aggressiveness. Although this assay provides a simple means to quantify aggression, the lack of physical contact between aggressors and targets limits its ability to mimic natural fighting behaviors. Interestingly, live fish have not been used as targets in this assay setup. Instead, when two fish interact through a transparent window, their behaviors were typically interpreted as social interaction (such as in a two-compartment social preference assay) rather than aggression.

Dyadic fighting assays examine aggression in a more natural setting. Although fighting behaviors are highly complex, stereotypical bouts can be repeatedly observed throughout a fight (Teles and Oliveira, 2016b; Zabegalov et al., 2019). Traditionally, a human observer monitors the process, manually annotates these behavioral bouts and keeps track of the outcomes of a fight (Chou et al., 2016). Alternatively, a recently developed analysis pipeline automatically annotates stereotypical fighting behavior with sub-second precision (Laan et al., 2018) (Fig. 3C), demonstrating great promise in applying unsupervised machine learning methods to studying complex natural behaviors.

The social hierarchy of a group can be assessed from dyadic fighting outcomes. Changes in social status have been associated with an individual's altered motor activity (Clements et al., 2018), reproductive success (Paull et al., 2010), and other physiological and health consequences (Filby et al., 2010a). Social animals adjust their behavior based on their status within a group, a phenomenon called social plasticity, which is also studied using aggression assays (Teles et al., 2016a; Maruska et al., 2019; Guayasamin et al., 2017; Sykes et al., 2018).

An animal can observe interactions between other individuals and use this information to adjust its own future behavior, a phenomenon named ‘social eavesdropping’. A common assay uses a two-chamber test arena divided by a one-way transparent window. An observer fish is placed in the chamber on the see-through side of the window, allowing it to observe the outcome of a fight in the opposing chamber without interacting with these fish (Abril-de-Abreu et al., 2015a). A video recording of a fight can also be used to train the observer fish (Abril-de-Abreu et al., 2015b). The time the observer fish spends in the vicinity of the observation window quantifies its attention. The observer fish remembers the participants and the outcome of the fight and adjusts its future dominant or submissive behaviors toward these individuals accordingly. Attentiveness toward a fight activates genes linked to neuronal plasticity, memory formation and alertness (Lopes et al., 2015).

As an assay with clearly separated binary outcomes, the aggression assay has been used to examine transcriptional (Malki et al., 2016; Oliveira et al., 2016) and neurophysiological (Teles and Oliveira, 2016a; Pavlidis et al., 2011; Filby et al., 2010b) outcomes of winning or losing a fight. Such effects were also examined in fish with different social statuses (Sneddon et al., 2011; Larson et al., 2006; Teles et al., 2016b).

### Mating

Highly stereotypical mating behaviors have been described for zebrafish (Darrow and Harris, 2004), although automated computer vision methods remain to be developed for mating behavior analysis. Several reports have created VR mimetics of the fish sailfin molly (*Poecilia latipinna*) and have used these animated animals to study the mating choices of live fish (Gierszewski et al., 2018, 2017; Müller et al., 2017a), an approach that may be transferrable to zebrafish. Zebrafish imprint visual and olfactory cues at 6 dpf, and use this phenotypic template to match and avoid mating with its own kin as adults (Gerlach et al., 2008; Hinz et al., 2013). Interestingly, exposure to non-kin cues does not result in successful imprinting (Gerlach et al., 2008), suggesting that additional mechanisms exist that regulate kin recognition (Biechl et al., 2016; Gerlach et al., 2019).

### Social learning

Zebrafish can learn from their peers. Social learning may help individuals in a group acquire public knowledge on resources such as food and threats such as predators without each individual paying the costs for learning. Naïve fish can learn an escape route (Lindeyer and Reader, 2010) or to find food (Zala and Määttänen, 2013) from a knowledgeable demonstrator fish. A separate study, however, reported that observer fish were unable to learn how to find food in a maze from demonstrators (Roy and Bhat, 2017). Wild-caught zebrafish are typically more timid than their domesticated counterparts. Wild zebrafish become bolder when exposed to domestic fish, without changing the level of boldness of the domestic fish, and this change in behavior persists after removal of the domesticated fish (Zala et al., 2012).

### Tracking individuals in a group using computer vision

To examine group dynamics during behavioral assays in more detail, researchers have explored methods to track individual fish in a group. This is a difficult task, as zebrafish swim in a 3D space, and individuals in a group will unavoidably cross over each other in a camera's view. Conventional solutions focus on deriving algorithms for predicting the trajectories of each fish. Before each crossover, the program calculates the most likely trajectory of each fish, so that, immediately after the two fish are separated, the algorithm assigns identities to each fish based on how well their new trajectories match the predictions. This method frequently introduces errors and unavoidably fails after long periods of tracking.

**Fig. 4.**
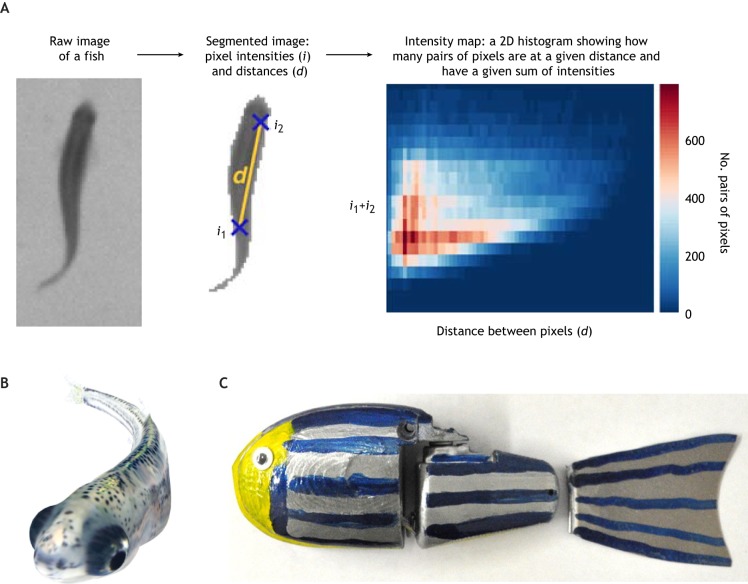
**Examples of emerging technologies for social behavior assays and analysis.** (A) idTracker for tracking individuals in a group. The raw images of each fish were first segmented to identify the body of each fish. Two pixels with intensities *i*_1_ and *i*_2_ are highlighted, which are separated by a distance, *d*. An intensity map is generated for each fish to show how many pairs of pixels are at a certain distance (*d*) and have a certain sum of intensities (*i*_1_+*i*_2_). This intensity map is used to identify each individual fish. Adapted with permission from Pérez-Escudero et al. (2014). This image is not published under the terms of the CC-BY licence of this article. For permission to reuse, please see Pérez-Escudero et al. (2014). (B) A virtual reality (VR) fish that mimics a 23-dpf real fish. Reproduced with permission from Stowers et al. (2017). This image is not published under the terms of the CC-BY licence of this article. For permission to reuse, please see Stowers et al. (2017). (C) A self-propelled robotic fish. Reproduced with permission from Butail et al. (2013a), where it was published under a CC-BY licence.

To solve this problem, the de Polavieja lab developed idTracker, which identifies and tracks individuals using a distinct digital fingerprint generated for each fish (Pérez-Escudero et al., 2014) (Fig. 4A). Crossover events still interfere with tracking, but only temporarily, as identities are reassigned after each crossover based on the fingerprint. This method enables researchers to acquire insights to previously difficult-to-observe behaviors in a group, such as territorial behavior. Their recently updated method, idTracker.ai, simultaneously tracks 100 individuals using deep learning with an impressive identification accuracy of greater than 99.9% (Romero-Ferrero et al., 2019). Building on the idTracker approach, several recent attempts have achieved multi-individual identification and tracking, with varying degrees of success (Bai et al., 2018; Qian and Chen, 2017; Qian et al., 2016, 2014; Wang et al., 2016a).

### Computational modeling of collective behaviors

Computational modeling has been applied to studying group behaviors of zebrafish and has generated valuable insights into group dynamics of fish and other species such as humans (Madirolas and de Polavieja, 2015). Previous methods largely ignored individual behaviors and focused primarily on examining static features of collective behavior at each time point, such as group cohesion calculated based on the distribution of individuals’ positions, and polarization, which is assessed by individuals’ orientations. This limitation may be attributed to limited understanding of stereotypic motions and difficulties in continuous tracking of individuals. Although ignoring individual identities allows each fish to be treated as a particle and aids the application of machine learning methods to characterize group behavior as a whole (Butail et al., 2013b), it inevitably limits further investigations on how individuals make behavioral decisions in a group.

One approach to overcome these limitations is to examine the behaviors of isolated individuals given different social cues, such as by using a three-chamber social preference setup (Fig. 3A) (Arganda et al., 2012; Porfiri and Ruiz Marín, 2017). Researchers have modeled individual behavioral rules in response to the motion of a social stimulus fish using theoretical frameworks based on the Bayesian decision theory (Box 2) (Arganda et al., 2012) and transfer entropy (Box 2) (Porfiri and Ruiz Marín, 2017). Other studies first improved continuous tracking of individuals and then computationally modeled pairwise interactions using the optimal control theory (Laan et al., 2017), deep attention networks (Heras et al., 2018), transfer entropy (Butail et al., 2016) and other data-driven methods (Zienkiewicz et al., 2018) to reveal how pairs of individuals attract, repulse and align with each other.

Recent studies have applied computational and machine learning methods to model individual stereotypical motions of *Caenorhabditis* *elegans* (Stephens et al., 2008; Brown et al., 2013), fruit flies (Berman et al., 2014), mice (Wiltschko et al., 2015) and zebrafish (Marques et al., 2018; Mwaffo et al., 2017; Zienkiewicz et al., 2015). Combining individual behavioral modeling with continuous individual tracking provides an opportunity to investigate group dynamics and individual decision-making with higher resolution. In one study, individual motions alternated between acceleration and deceleration bouts. The kinetics of these bouts could be described by sigmoid and exponential functions, respectively. Individual zebrafish motions were found to alternate between a ‘passive’ behavioral mode, in which behaviors of an individual are unaffected by other group members, and an ‘active’ mode, in which an individual’s behavior adjusts to social input from the group. This framework predicted behaviors of individuals with high precision (Harpaz et al., 2017).

### Virtual reality

Collective behaviors such as shoaling are mutual: each individual is driven by social cues emitted by its shoal mates and at the same time emits social signals that influence its shoal mates. Owing to the interactive nature of this closed-loop feedback system, it is difficult to disentangle a social input from the outputs it triggered. VR systems have the advantage of providing socially relevant inputs in a controlled and isolated manner. A projected virtual object moving in a way that mimics the characteristic kinetics of zebrafish swim bouts was sufficient to trigger shoaling of juvenile fish. Other previously implicated social cues, such as a fish-like shape or pigmentation pattern, were not required to trigger this behavior (Larsch and Baier, 2018). In another example, a virtual zebrafish was created to mimic a 23-dpf fish (Stowers et al., 2017) (Fig. 4B). In this interactive VR system, the movement trajectory of the virtual zebrafish was programmed to be influenced to varying degrees by the trajectory of a real fish. When set to be strongly influenced by the real fish, the virtual fish spent most of the time following the real fish and thus minimally affected the real fish's typical trajectory. Gradually reducing the level of social feedback resulted in the virtual fish exerting a stronger influence on the trajectory of the real fish, and therefore it seemed to ‘lead’ the real fish. Continued reduction in the degree of social feedback, however, eventually decreased the influence of the virtual fish on the real fish and led to its failure in leading.

### ‘Robot zebrafish’

Robotically controlled biomimetics have been developed to mimic animal behaviors and socially interact with animals such as fruit flies (Zabala et al., 2012) and cockroaches (Halloy et al., 2007), providing valuable insights into the social behaviors of these species. Similarly, a number of research groups have developed robotic fish (Cazenille et al., 2018). These systems are often composed of two parts: a biomimetic fish dummy and a robotic control system. The fish dummies can be directly fixed to a robot arm, indirectly linked to and moved by a robotic mechanism through magnetic coupling, or self-propelled (Butail et al., 2013a) (Fig. 4C), enabling them to ‘swim’ under water. Zebrafish respond to a robotically controlled dummy in a three-compartment social preference assay (Kopman et al., 2013; Ruberto et al., 2016, 2017). Compared to VR, a major advantage of robotic systems is their ability to provide physical contact between a biomimetic and an animal. Therefore, other groups have developed robot zebrafish that can come into physical contact with real zebrafish shoals (Cazenille et al., 2018; Polverino et al., 2012). However, the key features that allow a biomimetic fish to be socially integrated into a group of fish are still being debated. While many efforts focused on identifying socially attractive morphologies such as shape, size and pigmentation patterns for the dummy, other studies argued that a robot's behavior, such as its trajectories and movement kinetics, exert a greater influence on its ability to socially integrate into a shoal (Cazenille et al., 2018).

### Popular assays and their variations

Among the assays discussed above, the social preference, shoaling and aggression assays may be the most frequently used. Possible reasons for their popularity may be that they are relatively easy to set up, have intuitive relevance to social behaviors in humans, and have simple and quantifiable readouts. These assays are commonly used to assess changes in social behavior induced by disease-relevant treatments, as discussed further in the next section. Many variations exist for each assay, including but not limited to dimensions of the test platforms, numbers of animals used, types of stimulus (particularly for social preference and aggression assays), and quantification criteria and methods. This poses a potential challenge for the field, as the diversity of assays complicates interpretation of results and comparison between studies. It is worth noting that similar diversity is also widely present in rodent assays, which are currently still considered the gold standard for measuring social behavior. Nevertheless, efforts should be made to standardize current behavioral assay formats in zebrafish. Carefully designed experiments should be performed to evaluate these variations and provide recommendations for the optimal formats of each assay.

## Disease-relevant social-deficit models in zebrafish

In this section, we discuss recent advances in using zebrafish to model human social-behavior-related disorders. Although non-behavioral endpoints exist, including anatomical changes and endophenotypes, behavioral assays most directly demonstrate the relevance of these models to actual human behavioral disorders. Therefore, we focus on studies that model these disorders using social behavior assays. We categorize these studies based on the different methods used to induce social deficits.

### Genetic models

Technologies such as CRISPR, transcription activator-like effector nucleases (TALENs) and zinc-finger nucleases (ZFNs) have been implemented in zebrafish to generate genetic models. Forward-genetics methods can also generate mutants through random mutagenesis.

#### Autism risk genes

Modulating autism-related genes in zebrafish can induce autism-related phenotypes. However, the endpoints assessed in these studies have primarily focused on developmental and physiological changes or other comorbid behavioral symptoms of autism such as anxiety, sleep disorders and seizures. For example, *cntnap2* knockout induced night-time hyperactivity (Hoffman et al., 2016), and *chd8* morphants (Box 2) and mutants developed macrocephaly (Sugathan et al., 2014; Bernier et al., 2014). Researchers have started examining social behavior deficits in more recent studies. Knocking out the autism gene *shank3b* (Durand et al., 2007) induced deficits in shoaling, social preference and kin recognition (Liu et al., 2018a). Zebrafish with mutant *sam2*, ortholog to the human *FAM19A2* gene, were found to have shoaling (Choi et al., 2018) and social preference (Ariyasiri et al., 2019) deficits. The human *FAM19A2* gene is located in the 12q14.1 locus, home to a copy-number variation (CNV) associated with intellectual disability and autism (Autism Genome Project et al., 2007).

Zebrafish also demonstrated its rapid disease-modeling capability in a recent study in which a novel autism risk gene, *CEP41*, was identified by whole-exome sequencing. The zebrafish *CEP41* morphant showed deficits in social preference behavior (Patowary et al., 2019), providing experimental support for this new autism risk gene. A CRISPR-based targeted mutagenesis study systematically evaluated 35 autism and schizophrenia risk genes in an unsupervised machine learning assay for schooling (Tang et al., 2018). Significant behavioral changes were observed in the *immp2l* and *scn1lab* mutants; *immp2l* knockout enhanced shoaling, whereas heterozygous mutation in *scn1lab* seemed to suppress all evident social interactions between individuals. Their human ortholog, *IMMP2L*, is associated with Tourette syndrome (Petek et al., 2001), and *SCN1A* is associated with autism (Weiss et al., 2003) and Dravet syndrome (Wolff et al., 2006). Several other mutations also altered shoaling and schooling, but to a lesser degree.

#### Intellectual-disability risk genes

Intellectual disability is often comorbid with autism. Zebrafish knockout of *dyrk1aa*, an ortholog of the human Down syndrome gene *DYRK1A*, induced shoaling and social preference impairments (Kim et al., 2017b). Fragile X syndrome is a form of human intellectual disability caused by a loss-of-function mutation of the fragile X mental retardation 1 (*FMR1*) gene (Wu et al., 2017). Interestingly, knocking out of zebrafish *fmr1* caused precocious development of shoaling behavior, a phenomenon interpreted as a result of hyperactivity and increased anxiety (Wu et al., 2017), although such a phenomenon does not seem to be present in human patients with fragile X syndrome (Tranfaglia, 2011).

#### Schizophrenia risk genes

The zebrafish ortholog of the schizophrenia risk gene *DISC1* induced impaired shoaling response to stress when mutated (Eachus et al., 2017). Acute exposure to alarm substance (Box 2) or osmotic stress increased shoal cohesion in 5-dpf WT fish but not *disc1* mutants, suggesting its role in the development of the hypothalamic-pituitary-interrenal (HPI) axis, the fish equivalent of the hypothalamic-pituitary-adrenal (HPA) axis. Knocking out *adra1aa* and *adra1ab*, the two zebrafish orthologs of human *ADRA1A*, causes fish to freeze in tight groups for prolonged periods of time (Tang et al., 2018). Polymorphisms in the promoter region of the *ADRA1A* gene have been associated with schizophrenia (Clark et al., 2005), although not without controversies (Huang et al., 2008; Clark et al., 2006). While the freezing behavior found in fish is significant, whether or how this deficit translates to human disease phenotypes may require further investigation.

#### Other genetic models that cause social deficits

Researchers serendipitously discovered increased aggression in the *spiegeldanio* strain, an *fgfr1a^t3R705H/t3R705H^* mutant (Norton et al., 2011), during routine stock maintenance. This mutant showed increased mirror biting behavior and novel-object exploration, reminiscent of behavioral phenotypes seen in aggression-boldness syndrome. However, association of the human *FGFR1* with aggression has not been reported.

Leptin is generally known as an appetite regulator, but recent evidence has shown that it also plays roles in behavioral regulation (Morrison, 2009). Knockout of *lepa* by TALENs resulted in reduced aggression in a mirror-biting assay and reduced shoaling (Audira et al., 2018a). The authors argue that a dysregulated HPI/HPA axis may be responsible for the social deficit phenotype. In humans, an elevated leptin level has been associated with autism (Ashwood et al., 2008; Blardi et al., 2010; Raghavan et al., 2018) and Rett syndrome (Blardi et al., 2007, 2009), whereas a decrease in leptin is linked to schizophrenia and depression (Kraus et al., 2001; Atmaca et al., 2003).

### Gene expression modulation models

Changes in gene expression levels have been associated with social disorders. For example, CNV in chromosome region 16p11.2 is linked to autism (Sebat et al., 2007). Overexpression of the 29 genes encompassed by the 16p11.2 CNV in zebrafish identified *KCTD13* as an inducer of microcephaly (Golzio et al., 2012). Suppression of the same gene by morpholino resulted in macrocephaly (Golzio et al., 2012). Zebrafish morphants in several of these genes also showed deficits in brain-ventricle and midbrain development (Blaker-Lee et al., 2012). Although modulation of gene expression at the larval or adult stages is also possible using inducible expression systems (Chiu et al., 2016), this has not been utilized to establish social-deficit models in zebrafish.

### Chemically induced models: embryonic and maternal exposure

Both genes and the environment contribute to the development of social behavior. For example, environmental factors are estimated to account for 41% of autism risk (Gaugler et al., 2014). In fact, a number of environmental toxins, such as bisphenol A (BPA) (Stein et al., 2015), polychlorinated biphenyls (PCBs) (Lyall et al., 2017) and pesticides (von Ehrenstein et al., 2019), have been associated with elevated autism risk through epidemiological research, and were investigated in rodent models (Yu et al., 2011; Jolous-Jamshidi et al., 2010; Lan et al., 2017; Mullen et al., 2012). The zebrafish provides a powerful model for studying environmental factors that affect social development, especially given the simplicity of compound administration through water immersion. This subsection summarizes findings on how chemical exposure prior to or during embryonic development affects social behavior.

#### Alcohol and other abused drugs

Alcohol consumption during pregnancy can lead to fetal alcohol spectrum disorders (FASDs; Box 2). Patients with less-severe FASD can exhibit social deficits without anatomical changes (Seguin and Gerlai, 2018). Zebrafish embryos that were briefly (2 h) exposed to low levels (up to 1%) of alcohol develop to adults with no gross anatomical changes but show dose-dependent reductions in social preference to virtual (Fernandes and Gerlai, 2009) or live (Buske and Gerlai, 2011) social stimuli. This effect is likely mediated by impairments in the dopaminergic and serotoninergic systems (Fernandes et al., 2015; Buske and Gerlai, 2011). Embryonic exposure to another commonly abused drug, ketamine, did not significantly alter shoaling behavior (Félix et al., 2017a,b).

#### Prescription drugs

When taken during pregnancy, some common prescription drugs may have side effects or toxicity that affect the development of sociality in humans. In addition, due to their continuous usage and emission, pharmaceuticals often accumulate faster than they are removed from the environment and are considered pseudo-persistent contaminants (Mackay et al., 2014), making them accessible to humans through environmental exposure.

The effects of pharmaceuticals on social development can be conveniently modeled in zebrafish. For example, prenatal exposure to valproic acid can lead to fetal valproate syndrome (Box 2) in humans. Embryonic exposure to valproic acid or sodium valproate induced deficits in social preference behavior (Bailey et al., 2016; Baronio et al., 2018; Dwivedi et al., 2019; Zimmermann et al., 2015) but not aggression (Zimmermann et al., 2015). Impairments in the histaminergic (Baronio et al., 2018) and purinergic (Zimmermann et al., 2017) systems likely mediate this effect.

Embryonic exposure to 2 nM retinoic acid, an important signaling mediator in development, decreased social preference to a video of shoaling fish without inducing neural tube malformations or elevated death rate (Bailey et al., 2016).

Fluoroquinolones and tetracyclines are β-diketone antibiotics (DKAs) widely used in humans and animals. A 3-month exposure to a mixture of six DKA species, starting from birth, increased shoaling at a low concentration (6.25 mg/l) but inhibited shoal cohesion at a higher concentration (25 mg/l) (Wang et al., 2016b).

#### Environmental chemicals

Expansion of the chemical industry in the past century has greatly increased the number of environmental chemicals, yet only a fraction of these have been studied for their effects on the development of social behavior. Some commonly found environmental toxins have been studied in the zebrafish social behavior model. Benzo[α]pyrene often forms during organic-matter combustion and is found in cigarette smoke, diesel exhaust and grilled foods. When tested across three generations, benzo[α]pyrene induced a shoaling deficit in the first but not the subsequent generations (Knecht et al., 2017).

Chemical flame retardants are added to many household products. Among these, the brominated flame retardant (BFR) polybrominated diphenyl ethers (PBDEs) were widely used until the early 2000s. Although now largely phased out due to toxicity concerns, they persist in the environment, which can lead to continuous low-level exposure. When exposed to low doses of either of two prominent PBDEs, BDE-99 and BDE-47, zebrafish exposed to BDE-99 but not BDE-47 exhibited reduced social preference behavior (Glazer et al., 2018b). Another study reported elevated shoaling between pairs of zebrafish larvae following embryonic BDE-47 treatment (Zhang et al., 2017). Interestingly, the same group also tested two BDE-47 metabolites, 6-OH-BDE-47 and 6-MeO-BDE-47, using the same experimental setup, and found that 6-MeO-BDE-47 but not 6-OH-BDE-47 inhibited shoaling (Zhang et al., 2018). Another BFR, tetrabromobisphenol A, heightened aggression in males but not in females (Chen et al., 2016a).

BFRs have been largely replaced by a newer class of flame retardants, the organophosphate flame retardants (OPFRs). Currently, little is known about the potential developmental neurotoxicity of these chemicals. Six commonly used OPFRs showed no negative effect on shoaling behavior through embryonic exposure (Glazer et al., 2018a; Oliveri et al., 2015). A mixture of BFRs and OPFRs, FM 550 did, however, induce shoaling deficits (Bailey and Levin, 2015).

The organophosphorus pesticide dichlorvos (Altenhofen et al., 2019) and the neonicotinoid pesticide imidacloprid (Crosby et al., 2015) showed no effect on social behavior.

Endocrine-disrupting chemicals (EDCs) such as xenoestrogen have been suspected of affecting social behavior. Indeed, embryonic exposure to 17α-ethinylestradiol enhanced social preference behavior (Volkova et al., 2015).

BPA increased time spent near a mirror, but reduced male attacks on the mirror (Weber et al., 2015).

The heavy metals lead and arsenic were also tested, with lead exposure increasing aggression in a mirror test (Weber and Ghorai, 2013) and arsenic showing no effect on social behavior (Dipp et al., 2018).

#### Maternal exposure

Because humans develop *in utero*, environmental risk factors for social-behavior-related disorders must access the fetus through maternal exposure. Although exposure mechanisms in egg-laying fish and placental animals are significantly different, it is possible to model some aspects of this exposure mechanism in zebrafish. For example, exposing adult female zebrafish to a mixture of the water-soluble fraction of crude oil and lead was found to suppress shoaling behavior in their offspring (Wang et al., 2016c).

### Chemically induced models: adult exposure

Adult zebrafish can be used for pharmacological and toxicological studies. Drugs can be easily administered by direct water immersion, which enables drug absorption through the skin and gill, or oral ingestion. Both acute and chronic drug exposure can be conducted with good temporal control, as drugs can be added and removed at precise time points. To overcome potential issues in drug solubility and to enable pharmacokinetic analyses, drugs can also be applied by oral administration (Kulkarni et al., 2014; Dang et al., 2016) or intraperitoneal injection (Samaee et al., 2017). Drug absorption and metabolism can be measured by mass spectrometry (Villacrez et al., 2018).

#### Dietary components

Chronic exposure to dietary components can affect a body's nutritional and toxicological balance, which in turn modulate the overall health of an animal through regulation of metabolism and gene expression. Trace elements such as selenium and zinc are essential nutrients for mammals but are neurotoxic at excessive levels and their neurobehavioral effects on social behavior are not well understood. Chronic (60 days) exposure to selenomethionine, a naturally occurring selenoamino acid found in cereal grains, grassland legumes and soybeans (Whanger, 2002), suppressed shoaling in adult fish, potentially due to alterations of the serotonergic pathway (Attaran et al., 2019). Chronic (21 days) exposure to zinc chloride reduced mirror-biting behavior (Sarasamma et al., 2018). Hyperprolinemia is an inherited disorder of proline metabolism deficiency and has been associated with schizoaffective disorders (Jacquet et al., 2005; Orešič et al., 2011). To examine the effect of excess proline on social behavior, adult fish were exposed to 1.5 mM proline for 7 days. Impairments in social preference and other schizophrenia-related behaviors were found and rescued by the atypical antipsychotic drug sulpiride but not the typical antipsychotic haloperidol (Savio et al., 2012).

#### Environmental chemicals

Direct short-term (days) exposure to the herbicides glyphosate (Bridi et al., 2017) and atrazine (Schmidel et al., 2014) reduced aggressive behavior and shoaling, respectively, whereas an 18-day exposure to intraperitoneally injected paraquat did not significantly affect social interaction (Bortolotto et al., 2014). Acute exposure to gold resulted in a temporary reduction in social preference behavior that may be related to elevated oxidative stress; the social inhibition effect was short lived and the treated fish recovered within several hours (Strungaru et al., 2018). Chronic exposure to the EDC BPA reduced courtship behavior in females but increased their aggression towards mating competitors; females also preferred control males over BPA-treated males during courtship tests (Li et al., 2017a). Nonylphenol, another EDC and xenoestrogen compound, inhibited aggression and social preference behaviors by chronic exposure (Xia et al., 2010). 17α-ethinylestradiol, a synthetic estrogen and major component in oral contraceptive pills, is excreted from the human body in high amounts and accumulates in the environment. Its impact on zebrafish social behavior were examined in several studies to assess its influence on aquatic animals, revealing changes in social hierarchy and courtship in fish following exposure (Coe et al., 2008, 2009; Colman et al., 2009; Filby et al., 2012). Another EDC, triclosan, had inconsistent effects on social preference behavior (Liu et al., 2018b; Zang et al., 2019).

#### Neuroactive chemicals

Neuroactive chemicals have been applied to adult fish directly to investigate how different neurotransmitter pathways contribute to the regulation of social behavior and to examine a drug's therapeutic potential in treating social disorders. Oxytocin (OT) and arginine-vasopressin (AVP) are neuropeptides known to regulate social behavior in mammals. Their zebrafish homologs, isotocin (IT) and vasotocin (AVT), together with the mammalian OT and AVP, were examined in a social preference assay in which a WT test subject was placed between WT and *nacre* fish. Control- and vehicle-treated fish prefer to stay close to the WT social stimulus, whereas increasing doses of all four neuropeptides first reversed this preference, and then returned it to baseline, meaning that, at medium doses, the treated fish preferred to stay closer to the *nacre* than to the WT social stimulus (Braida et al., 2012). A synthetic oxytocin receptor ligand, dOTK_2_–C8, elicited a similar preference-reversal phenotype (Busnelli et al., 2016).

The dopaminergic system has been implicated in reward and social responses. Not surprisingly, the dopamine D1 receptor antagonist SCH23390 significantly reduced social preference in the WT AB zebrafish strain. Interestingly, researchers failed to observe a similar effect in another WT zebrafish strain, demonstrating natural variation in behavioral responses to neuroactive chemicals in different zebrafish strains (Scerbina et al., 2012). The common prescription drugs fluoxetine (Giacomini et al., 2016) and benzodiazepines (Giacomini et al., 2016; Schaefer et al., 2015) both inhibited shoaling. Fluoxetine also inhibited the offensive aggression behavior in dominant fish while suppressing freezing behavior in the subordinate fish (Theodoridi et al., 2017).

The glutamatergic N-methyl-d-aspartate receptor antagonist MK-801, commonly used to inhibit memory formation, has been used to create fish models with autism- and schizophrenia-like behavioral deficits. Acute exposure to MK-801 decreases social preference (Dreosti et al., 2015), shoaling (Maaswinkel et al., 2013a) and aggression (Zimmermann et al., 2016), an effect rescued by oxytocin and the oxytocin receptor agonist carbetocin (Zimmermann et al., 2016). Atypical antipsychotics sulpiride and olanzapine also reversed MK-801-induced social impairment, yet the typical antipsychotic haloperidol failed to reverse this phenotype (Seibt et al., 2011).

Nicotine significantly inhibits shoal cohesion but only mildly affects polarization, whereas ethanol strongly affects polarization within a fish school but only modestly inhibits shoal cohesion (Miller et al., 2013). In a social novelty test (Ariyasiri et al., 2019), control fish typically prefer to interact with a novel over a familiar fish. Ethanol exposure significantly suppressed this novelty preference behavior without affecting sociality in a three-chamber social preference test (Ariyasiri et al., 2019). Individual fish also respond differently to ethanol. While ‘shy’ individuals typically spent more time near a shoal than ‘bold’ fish, ethanol increased shoaling in bold fish but inhibited shoaling in shy fish (Araujo-Silva et al., 2018). The acute mild inhibitory effect of ethanol on sociality is enhanced by taurine, a common supplement in energy drinks (Fontana et al., 2018). Taurine also prevented alcohol-induced elevated aggression (Fontana et al., 2016). While acute ethanol exposure mildly inhibits shoaling, chronic (8 days) exposure to ethanol surprisingly increases shoal cohesion (Müller et al., 2017b). Chronic ethanol exposure dramatically lowered fertility when at least one of the mating partners was treated, and this inhibition was fully reversed by a 9-week withdrawal program (Dewari et al., 2016).

The psychotropic drug lysergic acid diethylamide (LSD) inhibited shoaling (Green et al., 2012; Grossman et al., 2010) but not social preference behavior (Grossman et al., 2010). Similar to the effects of oxytocin and arginine-vasopressin receptor agonists, the amphetamine derivatives 2,5-dimethoxy-4-bromo-amphetamine hydrobromide, para-methoxyamphetamine and 3,4-methylenedioxymethamphetamine generated an inverted-U-shaped curve in the *nacre*/WT social preference assay, shifting social preference from WT conspecifics to *nacre* and then back to WT fish at progressively increasing doses. Ketamine (Riehl et al., 2011) and ibogaine (Cachat et al., 2013) both inhibited group cohesion.

### Stressor-induced models

#### External stressors

Unpredictable chronic stress (UCS) and developmental social isolation (DSI) are often applied to animal models to mimic the environmental stressors that may contribute to psychiatric disorder development in humans. Zebrafish UCS assays apply different combinations of chronic stressors – such as restraint, social isolation, overcrowding, tank or water change, cold/heat, being chased by a net, dorsal body exposure in shallow water, exposure to air in a net, predator presence, and alarm substance – for varying durations (days to weeks). Stressors are often randomized on different days to ensure unpredictability. UCS assays with different stress protocols have generated inconsistent results, including increased (Chakravarty et al., 2013), first increased and then decreased (Piato et al., 2011), or unaltered (Fulcher et al., 2017) shoaling behavior after UCS. Acute stress by harassing the fish with a pen net prior to a behavioral test decreased social preference behavior but increased aggression (Giacomini et al., 2016). The effect of DSI on shoaling also remains controversial, as different reports have found it decreased (Shams et al., 2018) or did not alter (Fulcher et al., 2017) shoaling.

#### Physiological stressors

An induced inflammatory response by inoculating fish with formalin-inactivated *Aeromonas* *hydrophila* reduced social preference behavior (Kirsten et al., 2018), consistent with a previous report linking the immune system with social behavior in mice (Filiano et al., 2016). Traumatic brain injury (TBI) by pulsed, high-intensity focused ultrasound to the adult zebrafish brain increased shoaling cohesion (McCutcheon et al., 2017), although it may be difficult to determine the exact location and degree of brain damage caused by such a diffusive injury method. Hunger reduced aggression in females but not in males, possibly due to the females' stronger need to conserve energy compared to males (Ariyomo and Watt, 2015).

### Circuit manipulation models

Different parts of the subcortical social brain (SSB) play different roles in social behavior. Manipulating these brain regions and neural circuits through targeted neuronal inhibition, ablation and activation using genetic, optogenetic and chemogenetic (Box 2) approaches can help improve our understanding of the mechanisms regulating different aspects of social behavior. For example, targeted expression of tetanus neurotoxin to silence the lateral or medial subregion of the dorsal habenula (Table 1; Fig. 2) resulted in predispositions to lose or win a fight, respectively, revealing a dual control system for conflict resolution (Chou et al., 2016). In another study, manual (by inserting a 27½ G needle) and genetic ablations of a population of neurons in the ventral telencephalon inhibited social interactions, as quantified by failure to adjust orientation against a social stimulus fish (Stednitz et al., 2018). The ablated region is believed to be homologous to the mammalian lateral septum (Table 1; Fig. 2), a region implicated in social behavior in mammals (Clarke and File, 1982; Shin et al., 2018).

## Emerging technologies for modeling social behavior disorders in zebrafish: opportunities and challenges

In this section, we discuss emerging technologies that can potentially improve modeling of social behavior disorders in zebrafish. These can be broadly categorized as the ‘next generation’ methods for high-throughput model generation and drug testing, high-resolution functional brain imaging, and high-precision circuit manipulation for studying the circuit-level mechanisms of behavioral deficits. These research goals, limitations of current methodologies and potential solutions to overcome these limitations are summarized in Table 2.

**Table 2.**
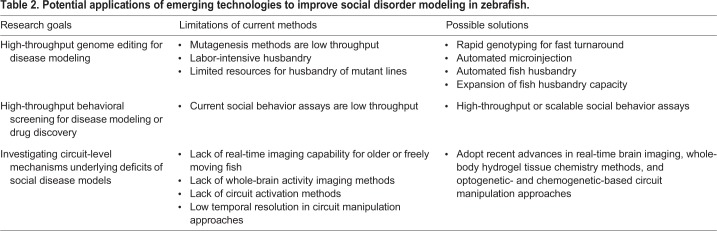
**Potential applications of emerging technologies to improve social disorder modeling in zebrafish.**

### High-throughput genome editing for disease modeling

Many neuropsychiatric disorders with social deficits have a strong genetic basis. Advanced genetic and genomic technologies have enabled researchers to find hundreds of genes that contribute to risks of developing neurological diseases. Given that the zebrafish is relatively inexpensive and easy to manipulate genetically compared to rodents, it has great potential as an experimental model to study these disease risk genes.

CRISPR is a popular technology for genome editing in zebrafish due to its simplicity and speed (Hwang et al., 2013; Prykhozhij et al., 2017; Prykhozhij and Berman, 2018). Researchers have attempted to improve throughput by developing more scalable methods. Current approaches are based on pooled CRISPR targeting followed by individual genotyping and separation, but have yet to provide a truly high-throughput output (Varshney et al., 2015; Shah et al., 2015). Several possible approaches may improve the current methods. Pooled CRISPR followed by early genotyping of live larvae using a recently developed approach (Lambert et al., 2018) can significantly speed up the turnover for each round of genotyping. Robotically controlled and fully automated embryonic injection methods (Zhao et al., 2018) also have the potential to increase the throughput of CRISPR delivery. Automated feeding systems such as Tritone (Aquatic Solutions) may facilitate the husbandry of large numbers of mutant lines generated by high-throughput CRISPR editing. Finally, if large numbers of disease-related mutants are generated, expanding the capacity of zebrafish stock centers may be needed (Table 2).

### High-throughput chemical screening for disease modeling and drug discovery

Both genes and the environment contribute to the development of social behavior. The development of some social-related disorders is also believed to be affected by environmental factors such as prenatal exposure to certain chemicals. The zebrafish has been a popular model for *in vivo* chemical screening (Rennekamp and Peterson, 2015). Its *ex utero* development allows embryos to be exposed to potentially toxic chemicals during early embryogenesis. The zebrafish larva is small, and a large number of larvae can fit into a compact imaging arena, enabling high-throughput behavioral profiling and phenotype-based drug discovery (Jordi et al., 2018; Bruni et al., 2016; Kokel et al., 2010, 2012; Rihel et al., 2010). These features make zebrafish an attractive model for systematically identifying potential environmental risk factors that contribute to disease etiology by high-throughput chemical and behavioral screening. Although technologies are readily available to expose zebrafish embryos, larvae or adults to chemicals in a high-throughput or scalable manner, a social behavior testing system capable of operating in a high-throughput or scalable fashion has yet to be developed (Table 2). The establishment of such a high-throughput social behavior assay in zebrafish would enable researchers to systematically screen chemicals for their disease-inducing risks and discover new drugs for treating social behavior deficits in humans.

### Neural activity imaging and circuit manipulation technologies for social disorder models

The circuit-level mechanisms underlying social behavior and disorders are not well understood. The zebrafish is a promising model for elucidating these mechanisms because, compared to rodents, it is relatively easy to image due to its small size and transparent nature. Furthermore, it is amenable to facile circuit manipulations through genetically targeted ablation, or optical and chemical activation or inhibition. Although many of these technologies are already established in zebrafish, they have rarely been used to investigate social behavior. Here, we discuss the potential to use imaging and circuit manipulation techniques to study brain activities in social behavior and disease models (Table 2).

Real-time calcium imaging has long been applied to larval zebrafish less than a week old. Researchers recently developed a two-photon calcium imaging approach that enables real-time brain imaging of 3-week-old zebrafish (Jetti et al., 2014; Vendrell-Llopis and Yaksi, 2015), a stage when robust social preference behavior is developed. Although, in the current methods, the fish must be restrained for imaging, combining this method with VR technology may allow brain activity imaging during a virtual social interaction. In addition, with the recent advancements in real-time brain imaging of freely moving larvae (Kim et al., 2017a; Cong et al., 2017; Muto and Kawakami, 2016), it may one day be possible to develop calcium imaging methods for freely moving 3-week-old fish during physical social interactions. Alternatively, whole-brain or whole-body tissue clearing methods such as CLARITY and PACT have been successfully applied to adult zebrafish (Cronan et al., 2015), and could enable post-hoc analysis of whole-brain activity patterns during social interactions by examining the expression of immediate early genes (Box 2).

Conventional methods in zebrafish manipulate circuit activity by inhibiting neuronal activity through expression of neurotoxins such as tetanus or botulinum toxins, or ablation of targeted neurons by expressing nitroreductase (Curado et al., 2008). Newer technologies such as optogenetics (Förster et al., 2017) and chemogenetics (Chen et al., 2016b) (Box 2) can be applied to zebrafish to activate or inhibit certain brain regions. It is also possible to combine both methods by applying an optically switchable compound to activate certain neurons (Lam et al., 2017). These newer methods can activate specific neurons with reversible temporal control.

## Conclusions

Animal models for behavioral disorders are inevitably confronted with questions about their validity. This is true for emerging model organisms such as the zebrafish as well as for established ones such as rodents. Care must be taken when interpreting results acquired from animal models that attempt to mimic symptoms of human conditions. This is especially true for complex traits such as social behaviors. Nevertheless, behavioral assays often provide readouts that are more relevant to the core symptoms of human behavior disorders than other assays that examine changes in anatomy, physiology or endophenotypes. Therefore, efforts in advancing the current assay and analysis methods of social behavior are necessary to facilitate progress in disease research.

The extensive data discussed in this Review support the idea that many of the most fundamental elements of social behavior – e.g. conspecific association, communication, establishment of hierarchies, social eavesdropping, aggression and mating – are conserved in social vertebrates from teleosts to mammals. Vertebrates as evolutionarily distant as zebrafish and rodents share numerous genetic, pharmacological, neuroanatomical and behavioral similarities relevant to social behavior. Common structures in the SSB may provide a biological foundation for social behavior conservation among vertebrates. We argue that the more complex, higher-order social behaviors in humans must be understood as layered on top of the SSB, and studying the zebrafish SSB can therefore have direct implications for understanding sociality in humans. Given the numerous methods currently available for studying various aspects of social behavior in zebrafish, the existing zebrafish models of social deficits and the technologies (established and emergent) for high-throughput experimentation, we anticipate that the zebrafish SSB will become an increasingly important model for understanding the biology of sociality in health and disease.
